# Novel prediction equations for appendicular skeletal muscle mass in hemodialysis patients: referenced against bioelectrical impedance analysis

**DOI:** 10.3389/fnut.2026.1735182

**Published:** 2026-04-23

**Authors:** Xinyu Wang, Lijuan Chen, Wenjing Yu, Dan Qiao, Li Li, Jian Wang, Bin Zhang, Zhiying Ang, Zhuqing Li, Ying Shen, Fei Chen, Yingchun Ma, Qinyuan Deng

**Affiliations:** 1 Department of Nephrology, Yunnan Province Spinal Cord Disease Clinical Medical Center, The First People’s Hospital of Yunnan Province, Kunming University of Science and Technology Affiliated Hospital, Kunming, China; 2 Department of Nephrology, The First People’s Hospital of Yunnan Province, School of Medicine, Kunming University of Science and Technology, Kunming, China; 3 Department of General Practice, Huaning People's Hospital, Yuxi, China; 4 Department of Nephrology, Beijing Boai Hospital, China Rehabilitation Research Center; School of Rehabilitation Medicine, Capital Medical University, Beijing, China

**Keywords:** appendicular skeletal muscle mass, bioelectrical impedance analysis, hemodialysis, prediction equations, sarcopenia

## Abstract

**Background:**

Accurate assessment of appendicular skeletal muscle mass (ASM) is essential in clinical practice and research involving patients undergoing hemodialysis (HD). However, ASM prediction equations developed in the general population may be inappropriate for HD patients because of dialysis-related alterations in body composition and hydration status.

**Objective:**

To evaluate the performance of existing anthropometric ASM prediction equations in HD patients and to develop dialysis-specific equations tailored to this population.

**Methods:**

In this cross-sectional study, 111 patients receiving maintenance hemodialysis were enrolled. ASM was measured using multi-frequency bioelectrical impedance analysis (BIA) under standardized post-dialysis conditions. The performance of three previously published equations, namely the height–weight (HW), limb-length–circumference (LC), and height–circumference (HC) models, was assessed. New dialysis-specific equations were developed using anthropometric variables through linear regression. Agreement was evaluated using Bland–Altman analysis, and internal validation was performed using cross-validation and bootstrap resampling.

**Results:**

Existing equations demonstrated limited agreement with BIA-measured ASM, showing substantial systematic bias and wide limits of agreement. Two dialysis-specific equations were developed: an advanced HW model (adjusted R^2^ = 0.785, RMSE = 2.712 kg) and a HW plus HC (HH) model (adjusted R^2^ = 0.807, RMSE = 2.628 kg). Both models showed markedly improved agreement with minimal bias and narrower limits of agreement compared with the original equations. Internal validation revealed more stable performance of the HH model, which maintained consistent agreement across internal validation and ultrafiltration-stratified sensitivity analyses.

**Conclusion:**

ASM prediction equations derived from the general population have limited applicability in patients undergoing maintenance hemodialysis. Dialysis-specific anthropometric calibration improves agreement with reference ASM, with the HH equation providing a practical and reliable tool for muscle mass assessment in this population.

## 1 Introduction

Sarcopenia, characterized by the progressive loss of skeletal muscle mass and function, is a significant predictor of increased morbidity, mortality, and reduced quality of life (1–4). Accurate assessment of muscle mass is therefore essential for nutritional and functional evaluation, particularly in vulnerable populations. Appendicular skeletal muscle mass (ASM) serves as a key operational measure for defining sarcopenia and is commonly estimated using prediction equations derived from anthropometric data in the general population (5, 6). Established models, such as those developed by Wen et al. for healthy Chinese adults, provide practical and non-invasive tools for ASM estimation in both epidemiological and clinical settings (7).

However, the direct application of these general population-based equations to hemodialysis (HD) patients is fundamentally problematic and is likely to yield invalid results. The unique pathophysiological environment of end-stage renal disease (ESRD), including chronic metabolic acidosis, systemic inflammation, uremic toxins, and frequent fluid shifts, alters the relationship between anthropometric indicators and true muscle mass, thereby interfering with standard body composition assessment (8). Moreover, ESRD-related muscle wasting follows distinct patterns compared with age-related sarcopenia, often involving disproportionate or region-specific muscle loss, which is not adequately captured by equations developed in healthy populations (9, 10). Consequently, when ASM prediction formulas validated in the general population are applied to HD patients, they may introduce systematic bias, leading to significant over- or underestimation of muscle mass (11).

The marked overestimation or underestimation of ASM by conventional equations may be explained by the distinct pathophysiological milieu of ESRD. Chronic inflammation, metabolic acidosis, and recurrent fluid shifts characteristic of maintenance hemodialysis (MHD) patients disrupt the associations between anthropometric measures and actual muscle mass (12–15). In addition, ESRD-related muscle wasting constitutes a distinct clinical entity from age-related sarcopenia, driven by uremic toxins, dialysis-related factors, and systemic inflammation rather than aging alone (16). These differences lead to distinctive patterns of muscle loss in ESRD that conventional equations from healthy cohorts fail to capture, highlighting the need for population-specific equations in chronic kidney disease (13, 17, 18).

Despite the urgent need for validated assessment tools, there is currently a notable lack of anthropometric equations specifically developed and validated for estimating ASM in maintenance hemodialysis patients. Therefore, the primary aim of this study is to evaluate the accuracy and applicability of existing ASM prediction equations developed for the general population in a cohort of hemodialysis patients, using bioelectrical impedance analysis (BIA) as the reference method. Furthermore, we seek to develop and validate novel, tailored anthropometric equations to accurately estimate ASM specifically within this unique patient population, thereby addressing a significant gap in the clinical management of sarcopenia in nephrology.

## 2 Methods

### 2.1 Study population and design

This cross-sectional study was conducted in a cohort of 111 patients undergoing MHD at the hemodialysis center of Yunnan First People’s Hospital. Participants were recruited from June 2025 to July 2025. Baseline anthropometric and clinical data were collected for all participants at enrollment. Written informed consent was obtained from each patient prior to enrollment. The study protocol was reviewed and approved by the Institutional Review Board of Yunnan First People’s Hospital (approval number: KHLL2025-KY051) and complied with the principles of the Declaration of Helsinki. Eligible participants were adults (≥18 years) who had received regular hemodialysis for at least 3 months. Patients with severe comorbidities such as active malignancy, acute infection, advanced cardiovascular disease, or severe musculoskeletal disorders that markedly interfere with the accuracy of anthropometric measurements (including advanced osteoarthritis with major joint deformity, severe rheumatoid arthritis, inflammatory myopathies, significant limb deformities or contractures, recent major fractures or orthopedic surgery, or neuromuscular disorders causing pronounced muscle atrophy), as well as those with limb amputation, were excluded. Severe renal osteodystrophy was not considered an exclusionary musculoskeletal disorder because it does not inherently prevent reliable anthropometric assessment.

### 2.2 Baseline data collection

Demographic and clinical data were obtained through medical record review and direct interviews. Standardized anthropometric measurements were performed by trained staff following established protocols. Height was measured to the nearest 0.1 cm using a stadiometer, and body weight was recorded in light clothing to the nearest 0.1 kg using a calibrated scale. Limb lengths (arm, upper arm, lower arm, leg, thigh, and calf) and circumferences (upper arm, forearm, thigh, and calf) were measured to the nearest 0.1 cm with a non-stretchable tape. Skinfold thickness at the triceps, thigh, calf, abdomen, and subscapular sites was assessed using a skinfold caliper (Harpenden, Baty International, UK, or equivalent). Waist and hip circumferences were also measured.

Ultrafiltration volume (L) during the hemodialysis session was obtained from dialysis treatment records. Anthropometric assessments were performed immediately after the hemodialysis session, following a 30-min supine rest period. Because all participants had an upper-limb arteriovenous fistula, measurements were obtained on the contralateral (non-access) arm. Data collection was conducted by three trained graduate students who received standardized instruction in anthropometric techniques, body positioning, equipment calibration, and procedural consistency, under the supervision of two physicians experienced in dialysis patient evaluation.

### 2.3 Reference ASM measurement

ASM was determined using a multi-frequency BIA device (model KBD500, Nanjing Cobetter Technology Co., Ltd., China). The device applies an 8-point tactile electrode method and measures impedance at three frequencies (5, 50, and 250 kHz) across five body segments (right arm, left arm, trunk, right leg, and left leg). Measurements were conducted under standardized conditions: post dialysis, with patients resting in a supine position for at least 30 min before assessment. The device operates at a measurement current of ≤200 μA and an impedance range of 100–750 *Ω*. In this study, the median ASM mass was 25.72 kg (interquartile range [IQR]: 22.12–30.39).

### 2.4 ASM prediction equations

Three anthropometric prediction equations developed by Wen et al. for Chinese adults were applied to estimate ASM (7). The first was the height–weight (HW) model: ASM = 0.193 × weight + 0.107 × height − 4.157 × gender − 0.037 × age − 2.631.

The second was the height–circumference (HC) model: ASM = height × (0.001509 × CAG^2^ + 0.0008555 × CTG^2^ + 0.0007709 × CCG^2^) − 4.044 × gender + 0.149 × weight − 0.038 × age + 12.246.

The third was the limb-length–circumference (LC) model: ASM = 0.000123 × ULL × CAG^2^ + 0.00002739 × TL × CTG^2^ + 0.0000269 × CL × CCG^2^–3.11 × gender + 0.128 × weight + 0.082 × height − 0.029 × age − 1.769.

In these equations, weight was expressed in kilograms (kg), height in meters (m), and age in years; gender was coded as 1 for men and 2 for women. ULL, TL, and CL represent upper limb, thigh, and calf length (cm), respectively. CAG, CTG, and CCG denote *corrected arm, thigh, and calf girths* (cm), calculated as follows:


$$\begin{array}{l} \text{Corrected circumference}=\text{measured circumference}-\pi \\ \times (\frac{\text{skinfold thickness}\;(\mathrm{mm})}{10}) \end{array}$$

That is,

CAG = upper arm circumference − *π* × (triceps skinfold ÷ 10),
CTG = thigh circumference − π × (thigh skinfold ÷ 10), and
CCG = calf circumference − π × (calf skinfold ÷ 10).

### 2.5 Sample size estimation

The primary objective of this study was to develop prediction models for ASM in patients undergoing maintenance hemodialysis, with ASM treated as a continuous outcome variable. Based on the planned multivariable regression framework, up to seven candidate predictors were considered. Following the commonly applied rule of at least 10 observations per predictor variable, a minimum of 70 participants was required for the training dataset. Given a predefined training set proportion of 0.85 and allowing for an estimated 10% rate of invalid or incomplete data, the minimum required total sample size was calculated to be 92 participants. For alternative model specifications involving four candidate predictors, the corresponding minimum sample size requirement was estimated to be 53 participants.

### 2.6 Statistical analysis

Continuous variables with a non-normal distribution were expressed as median (IQR), and categorical variables as numbers (percentages). Agreement between predicted ASM (derived from the three equations) and reference ASM measured by BIA was evaluated using Bland–Altman analysis, reporting mean bias, limits of agreement (LoA; mean bias ± 1.96 × SD of the differences), and the percentage of data points within the LoA. Correlations between variables were assessed using Spearman’s rank correlation coefficient (*ρ*). A two-tailed *p*-value <0.05 was considered statistically significant. Internal validation of the newly developed prediction equations was initially performed using five-fold cross-validation and a hold-out test set derived from the original cohort. Model performance was evaluated using the coefficient of determination (R^2^), root mean squared error (RMSE), mean squared error (MSE), and the Akaike information criterion (AIC), and learning curves were generated to assess model stability and potential overfitting. To further assess internal stability and minimize the influence of arbitrary data partitioning, bootstrap resampling was subsequently employed. Briefly, repeated bootstrap samples were drawn with replacement from the original cohort, and model performance was evaluated across resamples to estimate optimism and robustness. Performance metrics included R^2^, RMSE, mean absolute error (MAE), and bias, together with limits of agreement derived from Bland–Altman analysis. Bootstrap results are reported as mean values with corresponding variability. All analyses were performed using R software (version 4.2.1; R Foundation for Statistical Computing, Vienna, Austria).

## 3 Results

A total of 111 hemodialysis patients were enrolled in the study. The median age was 56.0 years (IQR: 47.0–66.0), and 60.36% were male (*n* = 67). The median ASM was 25.72 kg (IQR: 22.12–30.39). The median height was 1.65 m (IQR: 1.60–1.70). The median body weights were 60.70 kg (IQR: 51.90–67.75), with a median ultrafiltration volume of 2.5 L (IQR: 1.7–3.25). Median values for key anthropometric parameters included upper arm, forearm, thigh, calf, waist, and hip circumferences; arm, forearm, upper arm, leg, thigh, and calf lengths; and skinfold thicknesses measured at the triceps, subscapular, thigh, calf, and abdominal sites. Complete baseline characteristics are presented in Table 1.

**Table 1:** Baseline characteristics of the study population.

Variables	Total (*n* = 111)
Age (years), M (Q₁, Q₃)	56.00 (47.00, 66.00)
Height (m), M (Q₁, Q₃)	1.65 (1.60, 1.70)
Body weight (kg), M (Q₁, Q₃)	60.70 (51.90, 67.75)
BMI (kg/m^2^), M (Q₁, Q₃)	22.26 (20.01, 24.75)
ASM (kg), M (Q₁, Q₃)	25.72 (22.12, 30.39)
Ultrafiltration volume (L), M (Q₁, Q₃)	2.5 (1.7, 3.25)
Ultrafiltration <5% body weight, n (%)	81 (72.97)
Ultrafiltration ≥5% body weight, n (%)	30 (27.03)
Sex, n (%)	
Male	67 (60.36)
Female	44 (39.64)
Calf circumference (cm), M (Q₁, Q₃)	32.60 (30.35, 34.75)
Waist circumference (cm), M (Q₁, Q₃)	81.50 (75.50, 91.25)
Hip circumference (cm), M (Q₁, Q₃)	90.00 (86.70, 96.90)
Triceps skinfold (mm), M (Q₁, Q₃)	14.00 (10.00, 20.00)
Subscapular skinfold (mm), M (Q₁, Q₃)	17.00 (12.00, 22.00)
Thigh skinfold (mm), M (Q₁, Q₃)	14.00 (10.00, 20.00)
Calf skinfold (mm), M (Q₁, Q₃)	10.00 (7.50, 14.00)
Abdominal skinfold (mm), M (Q₁, Q₃)	20.00 (15.50, 28.00)
Upper arm circumference (cm), M (Q₁, Q₃)	24.00 (22.40, 26.70)
Forearm circumference (cm), M (Q₁, Q₃)	22.40 (20.65, 24.00)
Thigh circumference (cm), M (Q₁, Q₃)	42.80 (39.25, 45.25)
Arm length (cm), M (Q₁, Q₃)	69.50 (64.00, 72.65)
Upper arm length (cm), M (Q₁, Q₃)	33.00 (30.00, 34.30)
Forearm length (cm), M (Q₁, Q₃)	37.00 (34.00, 40.05)
Leg length (cm), M (Q₁, Q₃)	91.20 (87.00, 95.15)
Thigh length (cm), M (Q₁, Q₃)	47.60 (45.00, 50.50)
Calf length (cm), M (Q₁, Q₃)	43.90 (41.00, 46.50)

M, Median; Q₁, Q₃, First and third quartiles; BMI, Body mass index; ASM, Appendicular skeletal muscle mass.

**Figure 1:**
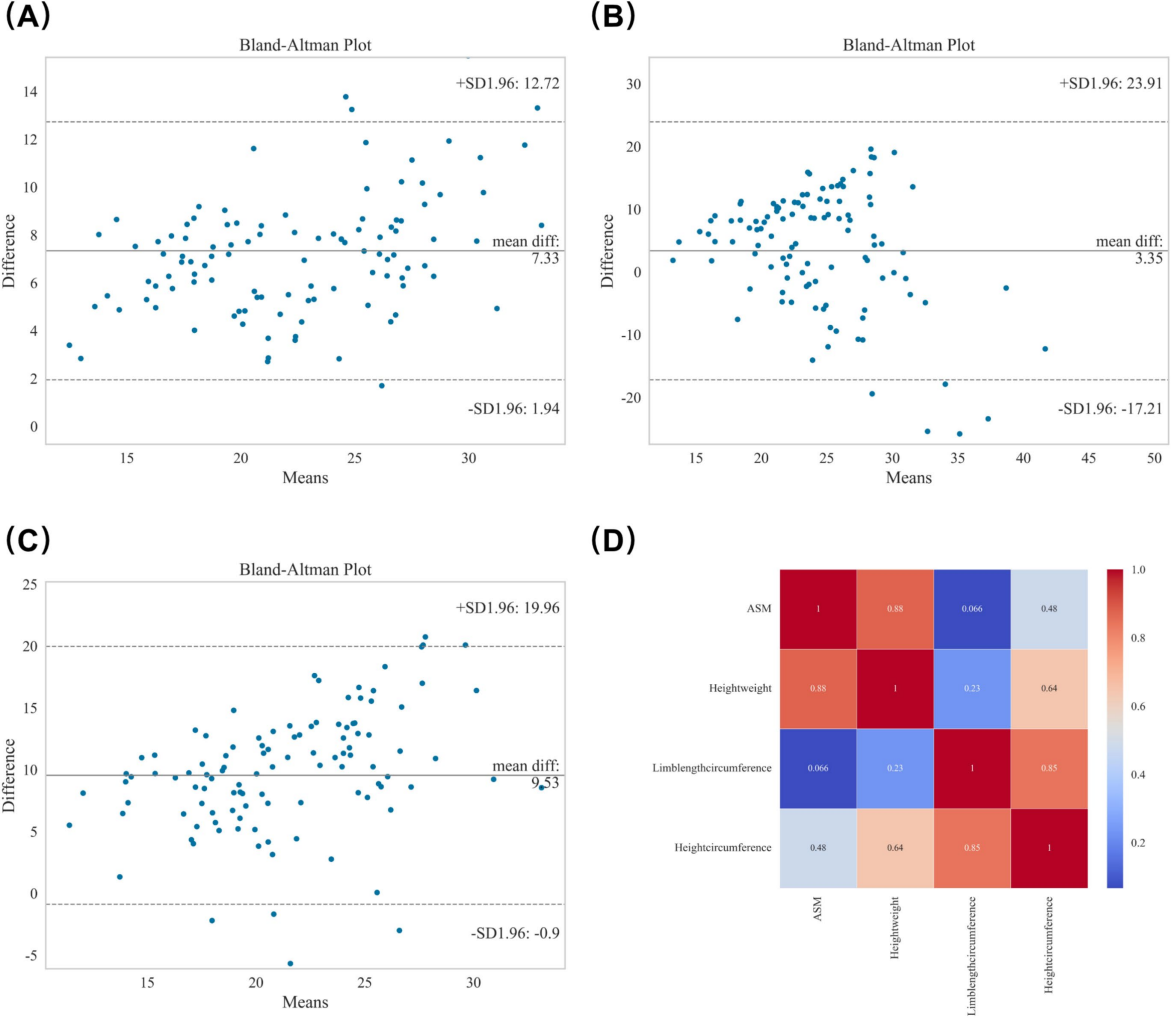
Agreement and correlation analyses of anthropometric equations for appendicular skeletal muscle (ASM) estimation. **(A)** Bland–Altman plot for the height–weight (HW) equation. **(B)** Bland–Altman plot for the limb length–circumference (LC) equation. **(C)** Bland–Altman plot for the height–circumference (HC) equation. **(D)** Spearman correlation analysis showing strong correlation for HW, moderate correlation for HC, and no significant correlation for LC with reference ASM; inter-equation correlations are also shown.

Agreement and correlation analyses of the original anthropometric equations with reference ASM are shown in Figure 1. In the Bland–Altman plots, the HW equation (Figure 1A) demonstrated a mean bias of 7.331 kg with limits of agreement from −12.722 to 12.722 kg, and 93.69% of the data points fell within these limits. The LC equation (Figure 1B) showed a mean bias of 3.349 kg with wider limits (−23.909 to 23.909 kg), with 94.60% of data points within the limits. The HC equation (Figure 1C) yielded a mean bias of 9.532 kg with limits from −19.961 to 19.961 kg, and 91.89% of data points lay within the range. Spearman correlation analysis (Figure 1D) demonstrated a strong correlation between the HW equation and reference ASM (*r* = 0.893, *p* < 0.001), no significant correlation for the LC equation (*r* = 0.101, *p* = 0.290), and a moderate correlation for the HC equation (r = 0.558, *p* < 0.001). Inter-equation correlations were HW vs. HC (*r* = 0.720, *p* < 0.001), HW vs. LC (r = 0.275, *p* = 0.004), and LC vs. HC (*r* = 0.774, *p* < 0.001).

**Figure 2:**
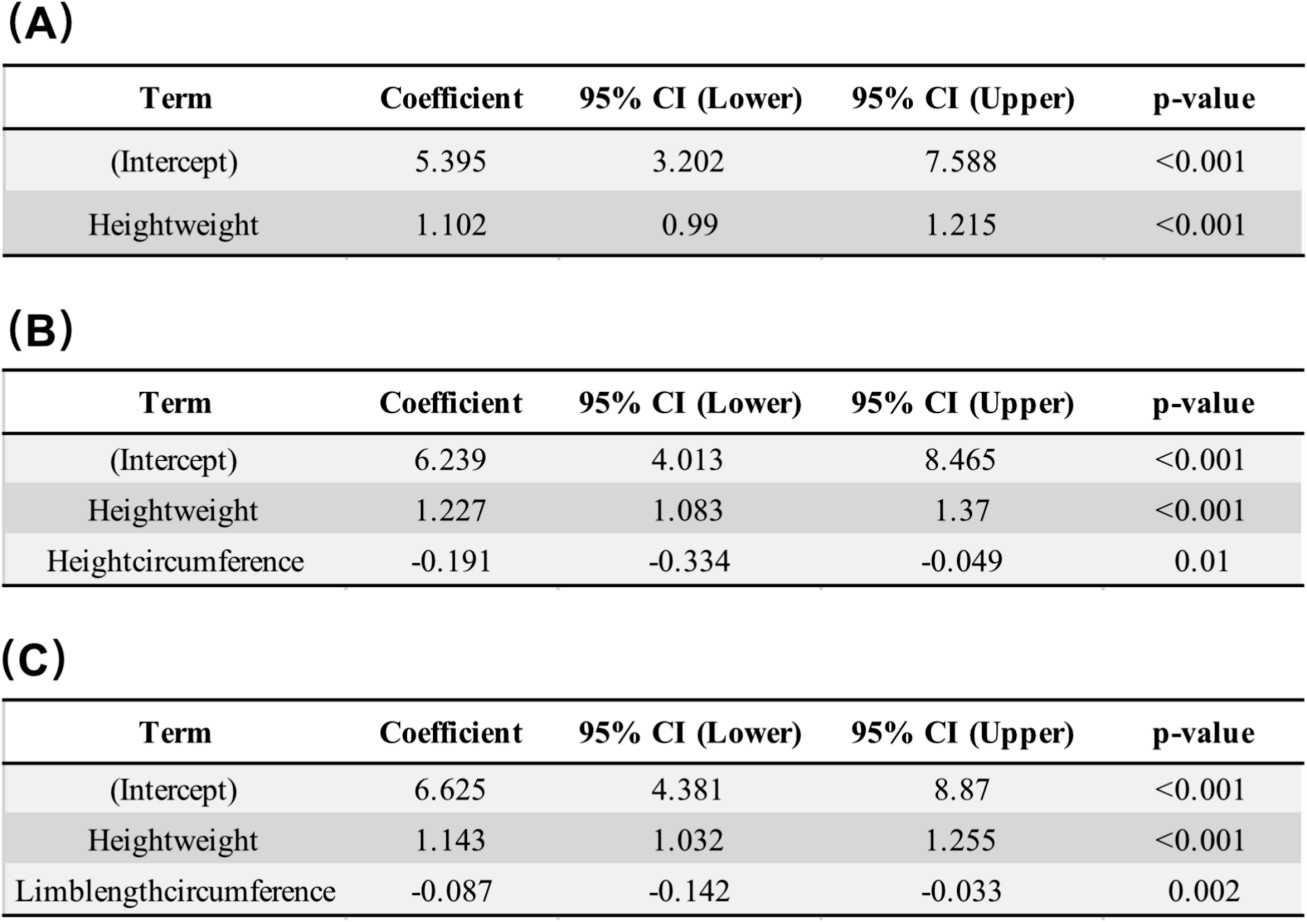
Development of modified regression equations for appendicular skeletal muscle (ASM) prediction. Three modified generalized linear regression models were developed: advanced HW, HH (HW + HC composite), and HL (HW + LC composite). **(A)** The advanced HW model (ASM = 5.395 + 1.102 × Height–weight) yielded an adjusted *R*^2^ of 0.785, RMSE of 2.712, and AIC of 542.462. **(B)** The HH model (ASM = 6.239 + 1.227 × Height–weight–0.191 × Height–circumference) showed superior performance, with an adjusted *R*^2^ of 0.807, RMSE of 2.628, and AIC of 537.540. **(C)** The HL model (ASM = 6.625 + 1.143 × Height–weight–0.087 × Limb length–circumference) demonstrated the best fit, with an adjusted *R*^2^ of 0.812, RMSE of 2.596, and AIC of 534.777. Residual analyses indicated acceptable goodness-of-fit for all three models.

To improve prediction accuracy, three modified regression models were developed using generalized linear regression (Figure 2). The advanced HW model (Figure 2A; ASM = 5.395 + 1.102 × HW) achieved an adjusted R^2^ of 0.785, RMSE of 2.712, and AIC of 542.462. The HW plus HC (HH) model (Figure 2B; ASM = 6.239 + 1.227 × HW – 0.191 × HC) showed superior performance, with an adjusted R^2^ of 0.807, RMSE of 2.628, and AIC of 537.540. The HW plus LC (HL) model (Figure 2C; ASM = 6.625 + 1.143 × HW – 0.087 × LC) demonstrated the best statistical fit in the training set, with an adjusted R^2^ of 0.812, RMSE of 2.596, and AIC of 534.777. Residual analyses confirmed acceptable goodness-of-fit for all three models.

**Figure 3:**
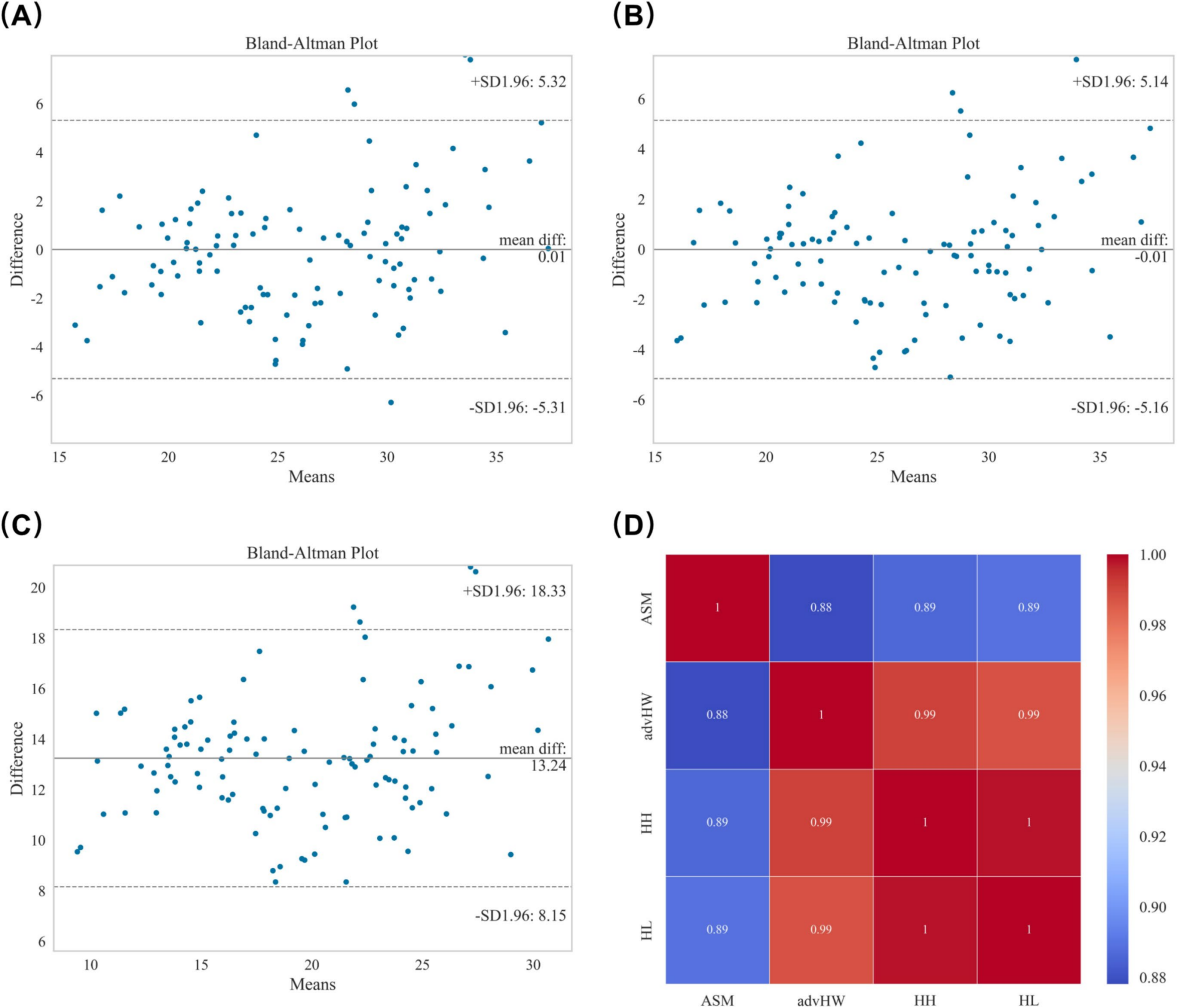
Agreement and correlation analyses of advanced regression models for ASM prediction. **(A–C)** Bland–Altman plots showing improved agreement of the advanced HW, HH, and HL models with reference ASM. **(D)** Spearman correlation analysis demonstrating strong correlations between all advanced models and reference ASM (*r* = 0.88–0.89, *p* < 0.001).

Agreement and correlation analyses of the modified regression models with reference ASM are presented in Figure 3. The advanced HW model (Figure 3A; ASM = 5.395 + 1.102 × HW) reduced the mean bias to 0.006 kg, with narrower limits of agreement (−5.321 to 5.321 kg), and 94.60% of data points lay within ±1.96 SD. The HH model (Figure 3B; ASM = 6.239 + 1.227 × HW – 0.191 × HC) yielded the smallest mean bias (−0.010 kg) with the narrowest limits (−5.142 to 5.142 kg), and 95.50% of points fell within these limits. In contrast, the HL model (Figure 3C; ASM = 6.625 + 1.143 × HW – 0.087 × LC) showed a large mean bias of 13.243 kg with limits from −18.331 to 18.331 kg; although 95.50% of data points were within ±1.96 SD, the pronounced systematic overestimation limited its practical utility. Spearman correlation analysis (Figure 3D) demonstrated strong correlations of all advanced models with reference ASM (*r* = 0.88–0.89, all *p* < 0.001), representing clear improvements compared with the original equations.

Considering the substantial systematic bias of the HL model, it was excluded from further validation. As an initial assessment of model fitting and sensitivity to data partitioning, the advanced HW and HH models were first evaluated using 5-fold cross-validation and a hold-out test set derived from the original cohort (training set *n* = 95; test set *n* = 16). In cross-validation, the advanced HW model (Supplementary Figure 1A) achieved a mean R^2^ of 0.765 ± 0.084 and a MSE of 7.778 ± 2.772, with a test-set R^2^ of 0.711 and MSE of 6.889. The HH model (Supplementary Figure 1B) yielded a mean cross-validation R^2^ of 0.772 ± 0.086 and mean MSE of 7.532 ± 2.758, and demonstrated a test-set R^2^ of 0.784 with MSE of 5.159. Learning curve analyses showed convergence between training and validation errors, suggesting no obvious overfitting under this resampling framework.

**Figure 4:**
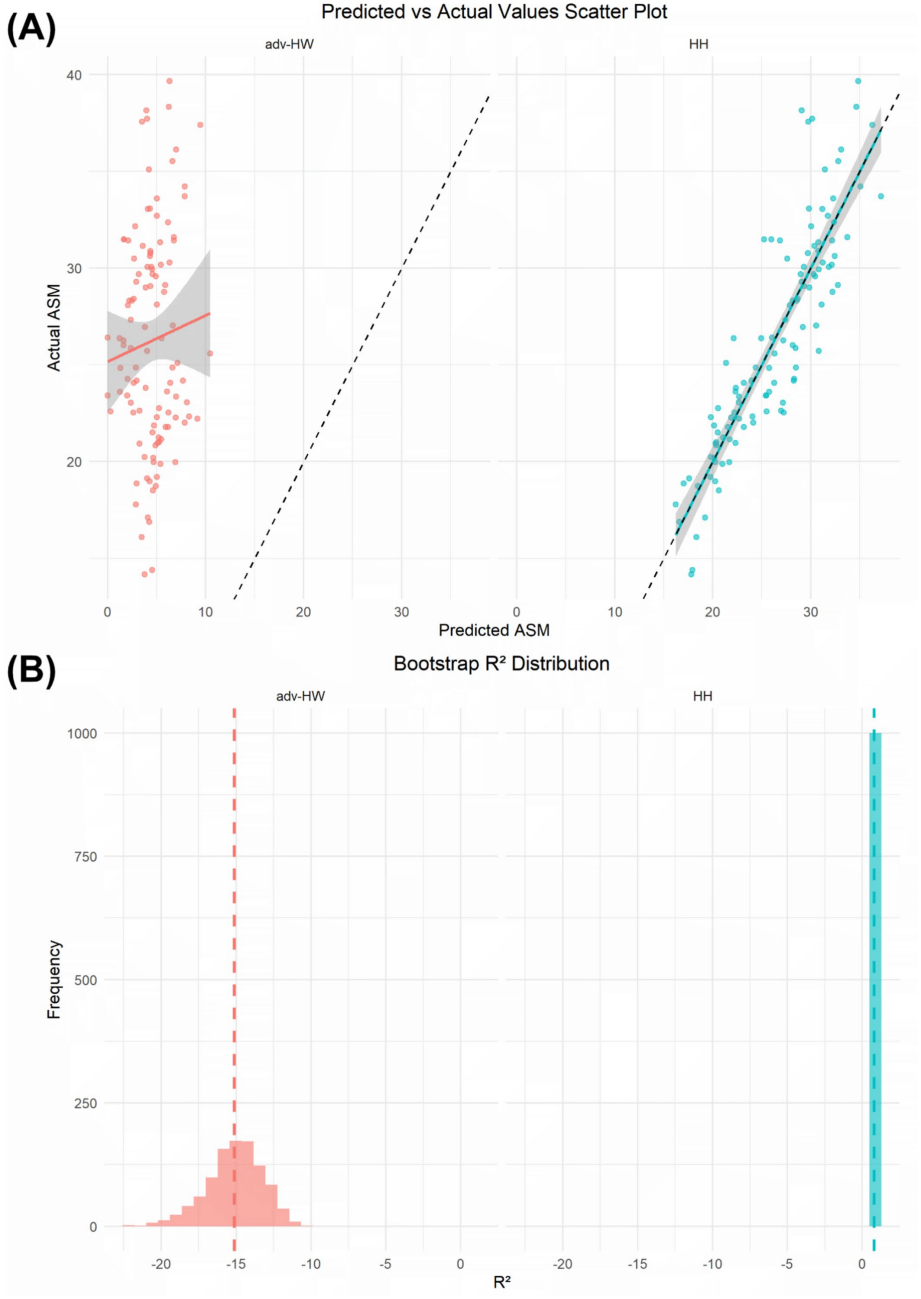
Internal validation of the modified anthropometric models. Predicted–observed scatter plots and bootstrap internal validation results for the advanced HW and HH models. **(A)** Scatter plots showing the relationship between predicted and reference ASM values for the advanced HW and HH models. **(B)** Bootstrap distributions of R^2^ values for the advanced HW and HH models based on repeated resampling. RMSE, MAE, bias, and limits of agreement are summarized for each model. Solid lines indicate the identity line in scatter plots. Bootstrap results are presented as mean R^2^ with corresponding variability.

To provide a more robust evaluation of internal stability and to minimize the influence of arbitrary data splitting, bootstrap resampling was subsequently employed as the primary internal validation strategy (Figure 4). Predicted–observed scatter plots revealed marked differences between the two models. For the advanced HW model, predicted ASM values were compressed within a narrow range despite wide dispersion of reference ASM values, resulting in substantial deviation from the identity line (Figure 4A). In contrast, predicted ASM values derived from the HH model closely tracked observed measurements, with data points distributed tightly along the identity line (Figure 4A). Bootstrap validation further highlighted model-specific differences in stability (Figure 4B). For the advanced HW model, the original R^2^ was −14.75, and the bootstrap mean R^2^ remained strongly negative (−15.10 ± 1.88), with a 95% confidence interval ranging from −19.22 to −11.88. Large prediction errors were observed (RMSE 22.49, MAE 21.71), together with pronounced systematic bias (−21.71) and wide limits of agreement (LoA − 33.24 to −10.19). In contrast, the HH model showed a stable bootstrap R^2^ distribution, with a mean R^2^ of 0.782 ± 0.035 (95% CI: 0.704 to 0.841), substantially smaller prediction errors (RMSE 2.63, MAE 1.95), minimal bias (0.01), and narrow limits of agreement (LoA − 5.17 to 5.18).

**Figure 5:**
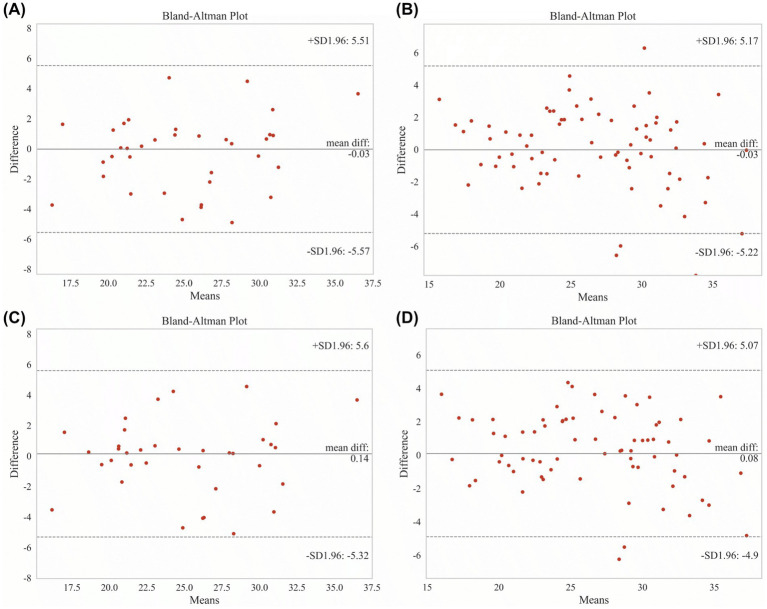
Sensitivity analysis stratified by ultrafiltration volume. Agreement analyses of the advanced HW and HH models with BIA-derived ASM after stratification by ultrafiltration volume relative to dry weight. **(A)** Bland–Altman plot for the advanced HW model in patients with ultrafiltration ≥5% of dry weight. **(B)** Bland–Altman plot for the advanced HW model in patients with ultrafiltration <5% of dry weight. **(C)** Bland–Altman plot for the HH model in patients with ultrafiltration ≥5% of dry weight. **(D)** Bland–Altman plot for the HH model in patients with ultrafiltration <5% of dry weight. Solid lines indicate mean bias, and dashed lines represent the limits of agreement (mean bias ±1.96 SD). Pearson correlation coefficients and the percentage of observations within the limits of agreement are shown in each panel.

Given the well-recognized influence of hydration status and fluid removal on body composition assessment in hemodialysis patients, sensitivity analyses stratified by ultrafiltration volume are shown in Figure 5. When patients were stratified according to ultrafiltration volume relative to dry weight (<5% vs. ≥ 5%), both the advanced HW model and HH models maintained good agreement with BIA-derived ASM. For the advanced HW model, strong correlations were observed in both the ultrafiltration ≥5% group (r = 0.846; Figure 5A) and the <5% group (r = 0.889; Figure 5B), with minimal mean bias (−0.032 kg and −0.025 kg, respectively), limits of agreement of ±5.511 kg and ±5.172 kg, and 97.30 and 91.89% of observations lying within ±1.96 SD. Similarly, the HH model demonstrated consistently high correlations in patients with ultrafiltration ≥5% (r = 0.853; Figure 5C) and <5% of dry weight (r = 0.899; Figure 5D), accompanied by negligible bias (0.138 kg and 0.084 kg), limits of agreement of ±5.596 kg and ±5.069 kg, and 97.30 and 94.60% of data points within the agreement limits. These findings indicate that the agreement of both models with BIA-measured ASM was preserved across different ultrafiltration strata, supporting their robustness against hydration-related variability.

In summary, among the three original anthropometric equations, the HW equation showed the strongest correlation and the most favorable agreement with reference ASM, whereas the LC equation showed no significant correlation and the HC equation demonstrated only moderate predictive performance. After regression-based modification, both the advanced HW and HH models improved agreement with reference ASM compared with the original equations. However, despite good apparent fit in the training set, the HL model showed substantial systematic overestimation and was excluded from further validation. Internal validation demonstrated clear differences between the remaining models: the HH model showed minimal bias, narrow limits of agreement, and stable performance across cross-validation, bootstrap resampling, and sensitivity analyses stratified by ultrafiltration volume, whereas the advanced HW model exhibited marked instability under bootstrap validation. Overall, these findings indicate that the HH model provides a reliable and clinically applicable approach for estimating ASM in hemodialysis patients.

## 4 Discussion

In this study, we systematically assessed the applicability of existing anthropometric equations for estimating appendicular skeletal muscle mass in patients undergoing maintenance hemodialysis and developed dialysis-specific prediction models. Equations originally derived from the general population showed limited agreement with BIA-measured ASM and notable systematic bias, underscoring their restricted transferability to the hemodialysis setting. In contrast, regression-based refinement substantially improved model performance, particularly for the HH model, which demonstrated minimal bias, narrow limits of agreement, and stable behavior across internal validation and ultrafiltration-stratified sensitivity analyses. These findings suggest that incorporating dialysis-relevant anthropometric information enhances the reliability of ASM estimation in this population, with the HH model representing the most robust approach among the evaluated equations.

Loss of skeletal muscle mass is highly clinically relevant in dialysis care because it contributes directly to protein-energy wasting, which is a major driver of hospitalization and mortality. Beyond its nutritional implications, muscle mass in hemodialysis patients is closely influenced by treatment-related physiological factors. For example, a recent study demonstrated that the skeletal muscle mass index decreases in patients with higher urea reduction ratios, indicating that dialysis adequacy and treatment-related catabolism play important roles in muscle preservation (19). Muscle quantity and quality also reflect broader aspects of patient health. Bioelectrical impedance analysis–derived phase angle is an integrated marker of cellular integrity, nutritional status, inflammation, and metabolic health. In hemodialysis patients, lower phase angle predicts protein-energy wasting (20), higher cardiovascular risk (21), frailty (22), and sex-specific thresholds have been proposed for older individuals (23). Consistent with these findings, a systematic review reported that nearly one quarter of dialysis patients have sarcopenia, and both low muscle mass and low muscle strength independently predict cardiovascular events and mortality (24). Functional impairments resulting from muscle loss are particularly important. Slow gait speed and weak handgrip strength predict mortality even more strongly than low muscle mass alone in dialysis populations (25), highlighting the multidimensional clinical relevance of skeletal muscle impairment in this population.

Reliable assessment of body composition in hemodialysis patients remains challenging because of chronic inflammation, fluid overload, and rapid fluid shifts. The 2021 European Society for Clinical Nutrition and Metabolism (ESPEN) Guideline reports that disturbances in hydration compromise the reliability of conventional methods such as dual-energy X-ray absorptiometry, computed tomography, and magnetic resonance imaging, and that these modalities are often not feasible for routine use in dialysis-dependent patients (26). Importantly, the guideline notes that bioelectrical impedance analysis is one of the few feasible bedside methods and that accuracy improves substantially when measurements are obtained after dialysis. This recommendation aligns with the 2020 Kidney Disease Outcomes Quality Initiative (KDOQI) Clinical Practice Guideline for Nutrition in CKD, which advises performing BIA or bioimpedance spectroscopy (BIS) at least 30 min after the dialysis session to minimize hydration-related variability (27). Emerging evidence indicates that muscle ultrasound has considerable potential as a bedside tool, showing good concordance with BIA-derived muscle measures and demonstrating usefulness for sarcopenia diagnosis in hemodialysis patients (28). However, despite this promise, current evidence and clinical experience suggest that BIA and anthropometric assessment remain the most widely applicable and feasible tools for routine practice, whereas imaging-based modalities such as DXA, CT, and MRI remain largely restricted to research settings due to high cost, limited accessibility, and poor practicality in routine dialysis workflows (10).

Efforts to maintain or increase muscle mass in dialysis patients further highlight the importance of practical monitoring tools. Nutritional intervention programs based on dialysis-specific dietary education have been shown to help maintain muscle mass and improve diet quality over time (29). Exercise interventions, particularly resistance training, have demonstrated significant benefits in improving skeletal muscle index, mid-arm muscle circumference, lean body mass, walking capacity, and functional performance among hemodialysis patients (30, 31). Some evidence suggests that combining exercise with nutritional support does not consistently augment improvements in body composition, although methodological heterogeneity limits firm conclusions (32). In addition, our own recently published work in a nationally representative cohort demonstrated that higher levels of physical activity are associated with better renal outcomes in middle-aged and older adults, further underscoring the broader significance of maintaining muscle health (33).

The divergent behavior of the advanced HW and HH models under different internal validation frameworks warrants further discussion. Although the advanced HW model showed acceptable performance in cross-validation and hold-out testing, it exhibited marked instability during bootstrap resampling. This likely reflects the limited robustness of a single composite predictor in hemodialysis patients, in whom body weight is strongly influenced by fluid shifts and extracellular water rather than skeletal muscle mass. Evidence from the Frequent Hemodialysis Network Trial indicates that changes in predialysis body weight largely reflect alterations in extracellular water rather than gains in body cell mass or muscle tissue, highlighting the limitations of weight-based indicators for muscle mass estimation in this population (34). In addition, split-sample and cross-validation approaches may yield unstable or optimistic performance estimates in relatively small datasets, whereas bootstrap resampling provides a more stringent assessment of internal validity. Methodological studies and contemporary guidance consistently recommend bootstrap validation as a preferred approach for internal model evaluation (35, 36). By contrast, the HH model integrates overall body size with circumference-based information that is more closely related to limb muscle structure and less sensitive to hydration-related variability, which likely explains its stable performance across bootstrap validation and ultrafiltration-stratified sensitivity analyses.

Existing ASM prediction equations were developed in healthy populations, and their coefficients therefore reflect anthropometric relationships that may not be preserved in patients undergoing hemodialysis (7). When applied to our cohort, these equations exhibited substantial systematic bias and wide limits of agreement, indicating limited suitability for the hemodialysis setting. In contrast, the equations proposed in this study were derived from anthropometric data obtained in hemodialysis patients under standardized post-dialysis conditions, allowing regression parameters to better capture population-specific body size and proportional characteristics. As a result, the newly developed models showed improved agreement with BIA-derived ASM. Notably, the HH model demonstrated stable performance across bootstrap internal validation and ultrafiltration-stratified sensitivity analyses, whereas the advanced HW model showed reduced robustness during resampling. Collectively, these findings support the value of dialysis-specific anthropometric calibration, with the HH model providing a more reliable and clinically practical approach for ASM estimation in maintenance hemodialysis patients.

Several limitations warrant consideration. The cross-sectional design limits causal inference and prevents evaluation of the prognostic implications of the new equations. The single-center sample may restrict generalizability, and future multicenter validation is needed. Although the new equations underwent internal validation using cross-validation and a hold-out test set, external validation in independent hemodialysis cohorts is still required to confirm their robustness and broader applicability. While BIA served as the reference method and measurements were standardized post-dialysis to minimize hydration-related variability, our device did not provide direct measures of hydration-related indices such as extracellular water to total body water ratio (ECW/TBW) or overhydration indices, which may further refine body composition assessment in hemodialysis patients. Finally, because the comparator equations were originally developed using dual-energy X-ray absorptiometry, further validation against imaging modalities or muscle ultrasound will strengthen confidence in their applicability.

## 5 Conclusion

Prediction equations for appendicular skeletal muscle mass derived from the general population show limited applicability in patients undergoing maintenance hemodialysis. In this study, two dialysis-specific anthropometry-based equations were developed and evaluated using BIA-derived ASM. Both models demonstrated improved agreement compared with existing equations; however, the HH model showed the most consistent performance, characterized by minimal bias, narrow limits of agreement, and stability across internal validation and ultrafiltration-stratified sensitivity analyses. These results support the value of dialysis-specific anthropometric calibration and suggest that the HH equation provides a practical and reliable approach for ASM estimation in the hemodialysis setting.

## Data Availability

The datasets presented in this study can be found in online repositories. The names of the repository/repositories and accession number(s) can be found in the article/Supplementary material.
