# Supplementary material for: Novel prediction equations for appendicular skeletal muscle mass in hemodialysis patients: referenced against bioelectrical impedance analysis
Source: Front Nutr. 2026 Apr 23;13:1735182. doi: 10.3389/fnut.2026.1735182 (PMC13149067; doi:10.3389/fnut.2026.1735182)
Supplement: Supplementary file 1 [file Table_1.DOCX]

**Supplementary Figure 1.** Internal validation of the HW and HH models.


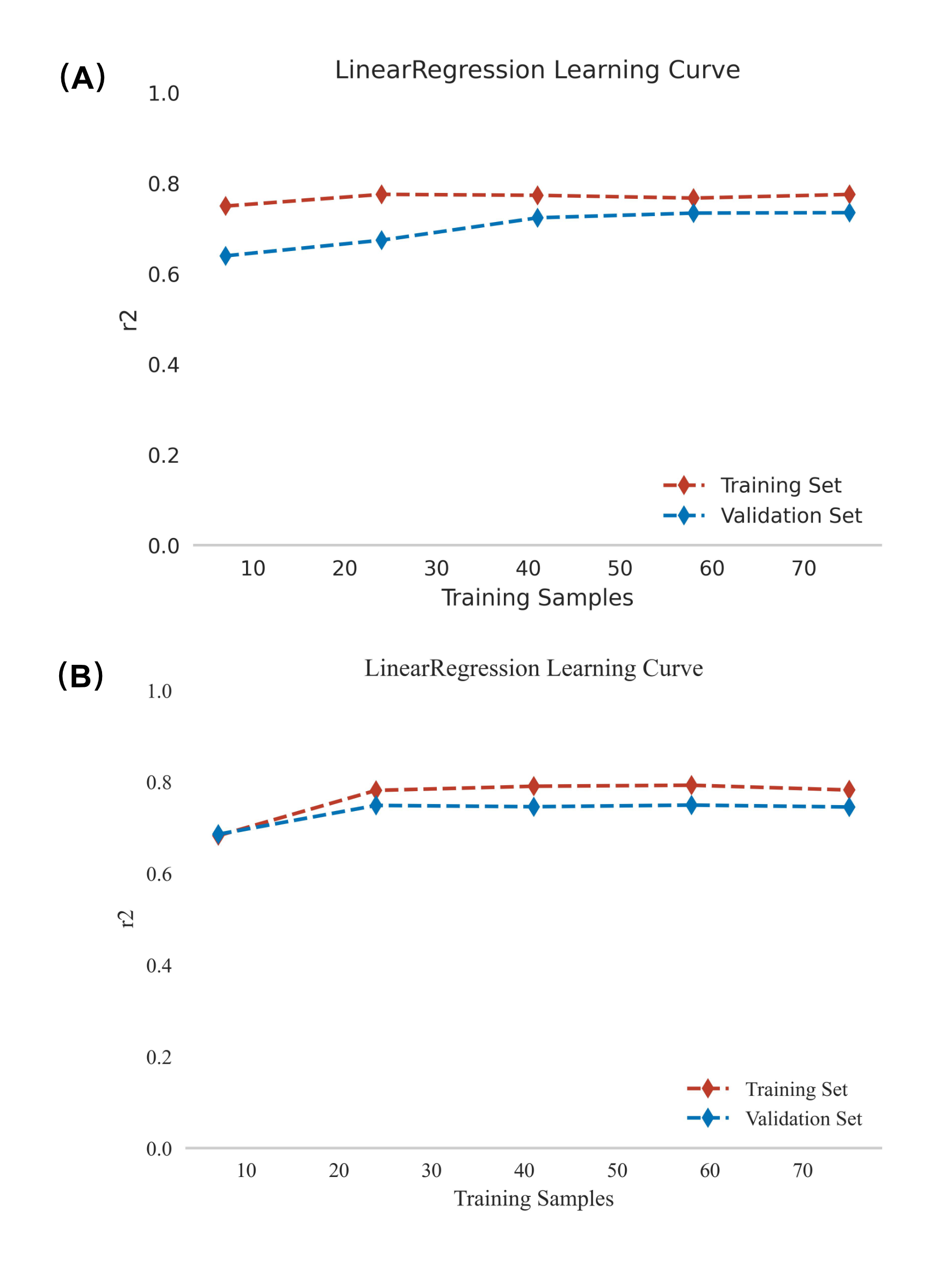


(A) Learning curve of the HW model, based on five-fold cross-validation and a hold-out test set, showing stable convergence between training and validation performance. The curve reflects consistent improvement in the coefficient of determination (R²) and mean squared error (MSE) without evidence of overfitting.
(B) Learning curve of the HH model, demonstrating superior and consistent generalization performance across cross-validation and test sets, with closely aligned R² and MSE trajectories indicating robust model stability.
